# Genomic Insights of Halophilic *Planococcus maritimus* SAMP MCC 3013 and Detail Investigation of Its Biosurfactant Production

**DOI:** 10.3389/fmicb.2019.00235

**Published:** 2019-02-26

**Authors:** Samadhan Waghmode, Mangesh Suryavanshi, Laxmikant Dama, Shraddha Kansara, Vikas Ghattargi, Parijat Das, Arun Banpurkar, Surekha K. Satpute

**Affiliations:** ^1^Department of Microbiology, Elphinstone College, University of Mumbai, Mumbai, India; ^2^National Centre for Microbial Resource, National Centre for Cell Science, Pune, India; ^3^Department of Zoology, DBF Dayanand College, University of Solapur, Solapur, India; ^4^Department of Microbiology, Savitribai Phule Pune University, Pune, India; ^5^Department of Physics, Savitribai Phule Pune University, Pune, India

**Keywords:** biosurfactant, halophile, *Planococcus*, terpene, genome sequencing, metabolite, surface tension

## Abstract

Moderate halophilic bacteria thrive in saline conditions and produce biosurfactant (BS) which facilitates the oil scavenging activity in the oil polluted surroundings. Production of such unusual bioactive molecules plays a vital role for their survival in an extreme and adverse environment. Current research deals with isolation of *Planococcus maritimus* strain SAMP MCC 3013 from Indian Arabian coastline sea water for BS production. The bacterium tolerated up to 2.7 M NaCl demonstrating osmotic stress bearable physiological systems. We used integrated approach to explore the genomic insight of the strain SAMP and displayed the presence of gene for BS biosynthesis. The genome analysis revealed this potential to be intrinsic to the strain. Preliminary screening techniques viz., surface tension (SFT), drop collapse (DC) and oil displacement (OD) showed SAMP MCC 3013 as a potent BS producer. BS reduced SFT of phosphate buffer saline (PBS) pH: 7.0 from 72 to 30 mN/m with a critical micelle concentration (CMC) value of 1.3 mg/mL. Subsequent investigation on chemical characterization, using thin layer chromatography (TLC), Fourier transform infrared spectroscopy (FT-IR), nuclear magnetic resonance (^1^H NMR and ^13^C NMR) and liquid chromatography mass spectrometry (LC-MS) revealed terpene containing BS having sugar, lipid moieties. The genomic sequence analysis of *P. maritimus* SAMP showed complete genes in the pathway for the synthesis of terpenoid. Probably terpenoid is the accountable backbone molecule for the BS production, but the later stages of terpenoid conversion to the BS could not be found. Moreover, it is important to highlight that till today; no single report documents the in-detailed physico-chemical characterization of BS from *Planococcus* sp. Based on genomic and functional properties, the term terpene containing BS is denoted for the surfactant produced by *P. maritimus*.

## Introduction

Halophiles are a type of extremophile organisms that thrive in high salt concentrations and produce novel metabolites which are not present elsewhere (Ma et al., 2010). Recently, marine natural products have gained comprehensive attention due to their extensive permanence under extreme environmental conditions (Karlapudi et al., 2018). Molecular profiling illustrates the occurrence of peculiar amino acid composition in their proteins enabling them to be folded at very high ionic strengths. This has been assumed to be the key mechanism by which halophiles shows adaptation in a high-salt containing surroundings (Tadeo et al., 2009). Hence, the marine ecosystem holds a great promise to discover the novel bioactive compounds like antibiotics, enzymes, drugs, vitamins, BS and many others (Bhatnagar and Kim, 2010; Antoniou et al., 2015; Waghmode et al., 2017).

The term ‘Biosurfactant’ (BS) represents surface-active molecules of microbial origin that exhibit mammoth structural and chemical diversity where glycolipids, phospholipids, lipopeptides, polysaccharide-protein complexes, fatty acids and neutral lipids are the popular one (Shekhar et al., 2015; Satpute et al., 2016). BS of marine origin demonstrates unique functional characteristics offering them to exploit for broad ranges of applications (Banat et al., 2000; Marchant and Banat, 2012). This ‘Green’ amphiphilic compounds are excreted out or may be cell bound (associated) facilitating microbes to utilize and accumulate between insoluble liquid phases through reducing surface tension (SFT) and interfacial tension (IFT) (Satpute et al., 2010a,Satpute et al., 2010b; Fakruddin, 2012). Thus, due to their extraordinary properties, microbial surfactant offer excellent candidature over the chemical surfactant for agriculture, environmental, food, cosmetics and petroleum industries (Gudiñna et al., 2015; Satpute et al., 2017,Satpute et al., 2018b).

Biosurfactants are structurally complex bio-molecules where diverse metabolic pathways are involved in their biosynthesis (Santos et al., 2016,Shaligram et al., 2016,Sun et al., 2018). For example, the bio-synthesis of lipopeptide type BS is mediated via nonribosomal peptide synthetases (NRPS). The NRPS are a kind of peptide secondary metabolites is also known in the synthesis of toxins, siderophores, pigments, antibiotics, etc. (Ansari et al., 2004,Satpute et al., 2010c,Song et al., 2015). Involvement of different genes and pathways ensures synthesis of various metabolites in microbes. Considerate knowledge of the genetic mechanisms involved in their biosynthesis would be helpful in expanding their economical scale of production. DNA sequencing technique is routinely used to sequence the whole genome or a region of microbial genome to reveal the pathways involved in their biosynthesis. Whole-genome sequencing (WGS) and transcriptome analysis facilitate molecular level profiling of desirable gene clusters where one can predict their transcriptional activity at any given time in the cell (Song et al., 2015). Thus, WGS of microorganisms holds huge promise for clinical diagnosis, public health and also for understanding the production mechanisms of microbial natural products (Kamada et al., 2014). Presently, microbial genome analysis is being vigorously performed by several researchers to understand their genetics, evolution, outbreak analysis, pathogenicity and antimicrobial resistance mechanisms (Edwards and Holt, 2013).

Genus *Planococcus* is a Gram-positive, non-motile halophilic bacterium which was proposed by Migula (1894). Despite earlier scientific studies on BS, the bacterium *Planococcus* with respect to BS cognition has remained largely unexplored for several years (Yoon et al., 2010). In the year of 2001, Engelhardt et al. (2001), isolated and characterized a novel hydrocarbon-degrading *P. alkanoclasticus* sp. nov. strain. Until the year 2007, there was no any detailed investigation on production of BS from *Planococcus* sp. The first report contributed by Kumar et al. (2007) documented the potential of *P*. *maitriensis* for BS/bioemulsifier (BE) production (without any structural details). Authors suggested that the production of EPS having composition of carbohydrate (12.06%), uronic acid (11%), protein (24.44%) and sulfate (3.03%). Later, the practical potential of *Planococcus* strain was identified for large scale BS production and oil cleansing potential (Ebrahimipour et al., 2014). These researchers reported the composition of *Planococcus* derived BS through gas chromatography (GC) and infrared (IR) spectroscopy studies. In 2015, Kavitha et al. (2015) also identified a new strain *P. jake* 01 and demonstrated its application for biogas production. Based on above literature, one can propose that the *Planococcus* derived BS certainly offers undeniable applications. In 2016, Ganapathy et al. (2016) stated methyl glucosyl-3,4-dehydro-apo-8-lycopenoate as a novel carotenoid, with antioxidant activity of *P. maritimus* origin. It is important to highlight that till today; no single report elucidates the structural and physical properties of BS from *Planococcus* origin. Indeed the need for genomics to search the metabolic capacity of well characterized bacterium along with functional metabolite would be helpful to develop mass production strategies. Thus, present investigation deals integrated approach of genomic insight of *P. maritimus* strain SAMP and the functional features of ‘terpene containing BS’ produced by the moderate halophile. To the best of our knowledge, possibly this is the first report on the draft genome sequence of *P. maritimus* SAMP, isolated from Indian Arabian coastline sea water. In addition, production of terpene containing BS with physico-chemical characterization has been included.

## Materials and Methods

### Sampling Locations and Isolation of Halophilic Bacteria

Seawater sample was collected in sterile polythene bag from the sea shore of Ratnagiri, Maharashtra, India (17.24° N, 73.37° E) and brought into the laboratory and maintained at 4°C for further use. The isolation of halophilic bacteria was carried out by using enrichment culture technique. Briefly, 1 mL of water sample was inoculated into 100 mL production medium with composition (per liter distilled water) 5 g yeast extract, 1 g (NH_4_)_2_SO_4_, 6 g Na_2_HPO_4_, 3 g KH_2_PO_4_, 2.7 g NaCl, 0.6 g MgSO_4_.7H_2_O and 2.0 mL trace element solution (TES) containing 10.95 g ZnSO_4_.7H_2_O, 5 g FeSO_4_.7H_2_O, 1.54 g MnSO_4_.7H_2_O, 0.39 g CuSO_4_.5H_2_O, 0.25 g CO(Na_3_)_2._6H_2_O, 0.17 g Na_2_B_4_O_7_.10H_2_O. The pH of the medium was adjusted to 7.0 and glucose (1.5% w/v) was used as a carbon source. The resulting medium was further incubated at 30°C on a rotary shaker (120 rpm) for 7 days. Following enrichment procedures, 1 mL of the culture sample was diluted and streaked on production medium agar plates containing glucose as a sole carbon source. The obtained bacterial colonies were further purified by streaking on nutrient agar plates (supplemented with 3% NaCl).

### Determination of Morphology of Bacteria

Scanning electron microscopy (SEM) was used to visualize the morphology of bacteria. The size and shape of the bacterium were recorded using instrument (Quanta 450 FEG, United States).

### Genomic Data Pre-processing, Assembly and Annotation

The genomic DNA of *P. maritimus* SAMP were extracted, purified and sequenced following the protocol described by Suryavanshi et al. (2017). The whole genome sequencing library was constructed using DNA kits (Nextera) and sequenced on the Illumina^®^ MiSeq platform. Quality of the genome sequence was analyzed for quality control with the help of FastQC software. The reads were trimmed and only the bases with quality above 20 (Q20) were used for assembly. After analysis, raw sequences were trimmed and assembled using *de novo* assemblers SPAdes 3.6.1 (Bankevich et al., 2012). The obtained contigs shorter than 200 bp were eliminated. NCBI-Prokaryotic Genome Annotation Pipeline was used to annotate and identify the metabolic pathways encoded on the draft genome. The ver3.3, released (2013) was used for annotation refinement and registration of the genome put at the International Nucleotide Sequence Database Collaboration (GenBank, United States) (Tatusova et al., 2016).

### Phylogenetic Analysis

The 16S rRNA gene from SAMP strain was directly fetched out from sequenced genome data. The obtained sequence was compared using the EZBioCloud Server and closely related sequences were retrieved. Further alignment was performed using ClustalW v2.0. Phylogenetic trees and bootstrap analysis were inferred following the procedures described by Zucchi et al. (2013). The tree was constructed with 1,370 bases of the 16S rRNA gene by using a neighbor-joining tree method with the Kimura 2-parameter and a 1,000 bootstrap for the confidence level (Kumar et al., 2016). CGview, a comparative genomics tool (Grant and Stothard, 2008) was used to visualize the genome of *P. maritimus* SAMP.

### Gene Prediction and Functional Annotation

The protein coding genes were envisaged with the help of Prodigal (Hyatt et al., 2010). Gene annotation was carried out with Rapid Annotation Subsystem Technology (RAST) using Prokka v1.11 (Seemann, 2014) and Prokaryotic Genome Automatic Annotation Pipeline (PGAAP) (Angiuoli et al., 2008). The genomic data were further screened for the presence of insertion sequence (IS) elements using, IS Finder (Siguier et al., 2006). IslandViewer4 tool was used to screen the genomic islands (GI) (Dhillon et al., 2015). The tRNA and rRNA genes prediction were performed using tRNAscan-SE v1.3.1 (Lowe and Eddy, 1997) and RNAmmer v1.2 (Lagesen et al., 2007) software. Specialized pipelines like antibiotics and secondary metabolite analysis SHell (antiSMASH) version 3.0.4 (Weber et al., 2015) and Natural Product Domain Seeker (NapDos) (Ziemert et al., 2012) were used to predict secondary metabolites.

### Whole Genome Comparison

Whole Genome sequences of *P. maritimus* MKU009 and *P. maritimus* DSM 17275 were downloaded from NCBI Genome database (Yoon et al., 2003; Ganapathy et al., 2016). These two and SAMP strain genomes collectively were RAST annotated individually. Comparative analysis of three genome sequences belonging to *P. maritimus* was done by an ultra-fast bacterial pan-genome analysis pipeline (BPGA) (Chaudhari et al., 2016).

### Data Submission

The *P. maritimus* strain SAMP was deposited at National Centre for Microbial Resource (NCMR) which formerly known as Microbial Culture Collection (MCC); National Centre for Cell Science (NCCS) Pune, under the accession number: MCC 3013. We have deposited whole-genome shotgun project at DDBJ/EMBL/GenBank under the BioProject ID PRJNA341789 with accession number MINM00000000. Integrated Microbial Genomes and Microbiomes (IMG/M) system for *P. maritimus* strain SAMP genome is available under taxon ID: 2808606941. The 16S rRNA gene sequence of SAMP strain was derived with the Sanger sequencing method and submitted to the GenBank under accession number: MH938046.

### Screening of SAMP for Biosurfactant Production

Initial screening of *P. maritimus* for BS production was carried out by SFT measurement, drop collapse (DC) and oil displacement (OD) techniques. All screening tests were performed as per the methods described by Satpute et al. (2008). SFT measurements were carried out using Optical Contact Angle Goniometer (For detail explanation see Physico-chemical characterization of BS in SFT measurement section). In DC test, the drop of culture supernatant (30 μL) was placed on a parafilm coated solid surface and observed for 1 min for drop collapsing. If the liquid contains surfactants, the drop spreads or collapses over a solid surface. The IFT between the liquid drop and the hydrophobic surface is reduced. In OD technique, 20 mL of distilled water was placed into empty petri dish followed by uniform layering of 20 μL of crude oil over the surface of the water. About 10 μL of cell free culture supernatant (CFS) was then added to the surface of oil. If BS is present in the CFS, the oil gets displaced showing the clear zone indicating the positive test.

### Production and Purification of Biosurfactant

The production of BS was performed in production medium supplemented with glucose (1.5% w/v) as sole carbon source. A seed culture (20 mL) was prepared by transferring a single colony of *Planococcus* from nutrient agar medium (supplemented with 3% NaCl). After 48 h of incubation, around 3% (v/v) of seed culture of *P. maritimus* was transferred as inoculum in a fresh fermentation media (150 mL) in 1 L Erlenmeyer flask. The complete production of BS was carried out at 30°C, 120 rpm for 7 days (Ebrahimipour et al., 2014). The production of BS was observed at 12 h intervals. Dried cell weight was measured in parallel to determine the biomass during the BS fermentation. Procedure carried out is as follows: Around 10 mL of culture broth was filtered through sterile filter paper (0.22 μm Millipore, Bengaluru, India), further dried at 105°C for 24 h and weighed. The dried biomass was weighed repeatedly till the constant weight was noted. BS fermentation process was carried out up to 7 days and the product was then extracted from cells free supernatant (CFS). All experiments were done in triplicate and the mean values were plotted along with the standard deviation (SD) using the GraphPad Prism 7.0 software (GraphPad, La Jolla, CA, United States). The CFB obtained by centrifugation, was subjected to acid precipitation (5 N HCl). The solvent system of ethyl acetate: methanol (4:1, v/v) was used for the extraction of BS at room temperature. The organic phase was subjected to rotary evaporation to obtain BS product (Hamza et al., 2017). The viscous honey colored BS was further purified by Silica gel (Merk, mesh size 60-120) column chromatography. The mobile phase of chloroform: methanol (100:0, 95:05, 90:10, 85:15 and 80:20) was used for elution. After elution different fractions were collected and analyzed using thin layer chromatography (TLC) (Satpute et al., 2010b).

### Physico-Chemical Characterization of Biosurfactant

#### Measurement of Surface (SFT) and Interfacial Tension (IFT)

In order to determine the critical micelle concentration (CMC) of BS, different concentrations (100–1,500 μg/mL) of purified product were prepared in sterile phosphate buffer saline (PBS). Ability of BS to reduce the SFT of PBS was determined by the pendant drop technique using Optical Contact Angle Goniometer (OCA 15 Plus, DataPhysics Instruments GmbH, Germany). The equilibrium shape of the pendant drop was captured using charge-coupled device (CCD) camera and analyzed in real time by using SCA 20 software. SFT values of each dilution were determined on the above instrument, and the CMC was determined by plotting values of SFT versus concentration of BS on semi-log scale. During the measurement, the instrument itself takes around 500 readings and the mean values of those 500 readings were displayed by the software which was taken for further analysis. The IFT of purified BS was measured at CMC value against the kerosene (purchased from local market) and 2T engine oil (HP racer, India). Detailed procedure is discussed in the recent publications of our group (Hamza et al., 2017).

#### Measurement of Contact Angle (CA) and Emulsification Activity

The contact angle (CA) of purified BS was determined at CMC solution on Teflon (highly hydrophobic), glass (highly hydrophilic) and parafilm (intermediate) as per the procedure described by Hamza et al. (2017). Emulsification properties of BS against various hydrophobic substrates like hexadecane, kerosene, toluene, coconut oil and castor oil were carried out as per the emulsification assay explained by Satpute et al. (2010b). Water and sodium dodecyl sulfate (SDS) (CMC solution 8 mM) were considered as negative and positive control, respectively.

### Stability Studies of Biosurfactant

The stability of purified BS was checked at different pH, temperatures and NaCl concentrations. Buffer solutions of different pH (2, 4, 6…14) were prepared and BS (1.3 mg/mL: CMC concentration) was added, incubated at room temperature for 12–24 h and subsequently SFT measurements were taken. Similarly, the SFT of BS was measured in a PBS (pH 7.0) and incubated at different temperatures (20, 40…and 121°C). SFT values were checked for saline solutions prepared with different NaCl concentrations (0, 5, 10…25%). SFT values of all three parameters (pH, temperature and NaCl concentrations) were measured before and after incubation time so as to determine stability of BS.

### Determination of Ionic Character

The ionic character of BS was determined by agar well diffusion method. Anionic surfactant such as SDS (20 mM), cationic surfactant cetyltrimethyl ammonium bromide (CTAB) (20 mM) and non-ionic surfactants Triton X-100 (0.9 mM) and Tween 80 (0.012 mM) were used for this study. Assay was performed as described by Diniz et al. (2014) and Satpute et al. (2018a).

### Analysis by Thin Layer Chromatography (TLC)

Thin layer chromatography was carried out for the detection of glycolipids, lipids, amino acids and sugars moieties. The TLC silica gel aluminum sheet (Si 60 F_254_, 0.25 mm, Merck KGaA, Germany) was activated by heating them on a heating plate (50°C) for few minutes before spotting. Samples were loaded and allowed to dry completely. Further plates were placed into solvent system of chloroform: methanol: glacial acetic acid, 65:25:02 (v/v/v). After the run, the plate was air dried and the spots were visualized by spraying with anisaldehyde reagent containing anisaldehyde: H_2_SO_4_: glacial acetic acid, 1:2:50 (v/v/v) and heating at 110°C for 5 min. Rhamnolipids (RHLs) (Agae Technologies, United States) was used as the reference. In order to determine presence of sugar and amino acid, the solvent system and developer were used as described by Jagtap et al. (2010) and Satpute et al. (2010b). The solvent and developing system of Wang et al. (2014) was used for lipid detection. The spots were visualized followed by noting their *R*_f_ values.

### Fourier-Transform Infra-Red (FT-IR) Spectroscopy

The chemical characterization of BS was performed using a FTIR spectrophotometer (PerkinElmer, United Kingdom). The analysis was done in the mid IR region of 500–4,000 cm^-1^ with 20 scan speed (Das et al., 2008).

### Nuclear Magnetic Resonance (NMR) Spectroscopy

The proton (^1^H and ^13^C) nuclear magnetic spectra were recorded at 298 K on a 400 and 101 MHz NMR spectrophotometer (Bruker, Germany). The samples were prepared as solutions in 100% CDCl_3_, using approximately 1–3 mg of the BS utilizing tetramethylsilane (TMS) as an internal standard (Pemmaraju et al., 2012).

### Liquid Chromatography-Mass Spectrometry (LC-MS/MS)

Ion electrospray mass spectra (ESI–MS) of BS were recorded on a Finnigan QTOF2 mass spectrometer (Thermo Quest LC and LC/MS Division, San Jose, CA, United States). Stock solution of the surface-active compound was prepared by dissolving of 2 mg substance in 1 mL chloroform/methanol (1:1, v/v). Aliquots of 0.1 mL were diluted in 1.9 mL acetonitrile/water (7:3, v/v) and infused at a flow rate of 10 μL min^-1^ with a syringe pump (Hamilton syringe, 500 μL) directly connected to the electrospray ionization (ESI). ESI mode was as follows: sheath gas-nitrogen (6l min^-1^); negative mode [M–H]– at -4 kV of ionization; the temperature and the voltage of the heated capillary were 300°C and 25 V, respectively; tube lens offset 5 V. Helium was used as a collision gas at the collision-induced dissociation (Hu and Ju, 2001).

## Results

For bio-prospecting of marine bacteria, the surface bio-active compounds such as BS is explored from indigenous halophilic bacteria. Identification of marine bacterium and the detailed investigation on physical properties like SFT, CMC, IFT, EI and stability studies are supportive to prove the surfactant properties of microbial originated surface active compounds.

### Microscopic Observation and Identification

**FIGURE 1:**
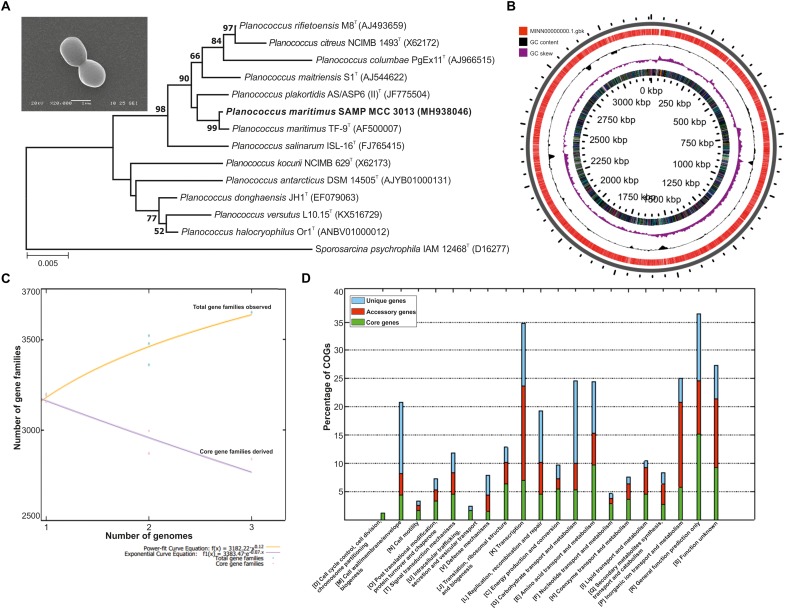
**(A)** Phylogenetic tree reconstructed by a neighbor-joining analysis based on 16S rRNA gene sequences, depicting the phylogenetic relationship of strain SAMP to related type strains of the genus *Planococcus* Bar, 0.005 changes per nucleotide position. Bootstrap values (%) >50% from 1,000 replicates are shown and inset showing scanning electron micrograph (SEM) of *P. maritimus* SAMP MCC 3013. **(B)** Circular genome plots. The outermost concentric circles denote the predicted protein-coding genes present in both forward strand and the reverse strand. The next concentric represents the GC content and the purple color show the GC content below average. **(C)** The numbers of shared genes were plotted as a function of number of strains. **(D)** COG distribution in core, accessory and unique genomes.

The morphological traits of isolated bacterium were identified using SEM and are 1.4 μm in size and possess short cocci shaped morphology. The identification of the isolates on the basis of 16S rRNA disclosed it is a phylogenetic neighbor of *P. maritimus* (Figure 1A) with 99% identity; as performed at EzBiocloud.

### General Genome Information

The whole genome of *P. maritimus* SAMP was sequenced using the Illumina MiSeq sequencing platform. A total of 3,251,644 Illumina reads were assembled using SPAdes genome assembler version SPAdes v. 3.6.1, which resulted in 43 contigs with an N50 value of 2,065,493 bp. The *de novo* genome assembly of strain SAMP resulted in 43 scaffolds with a genome size of 3,220,000 bp and G+C content of 47.2%. A sum of 3,111 protein-coding sequences (CDSs) was predicted in the genome, out of 93 copies of RNA: 26 rRNA, 63 tRNAs and 4 ncRNA were identified and the circular view is shown in Figure 1B.

### Pan-Genome Analysis

The pan-genome analysis was done by using available genomes from the NCBI genome database. The general genome features for all the three strains are shown in Table 1. The pan-genome analysis suggested the presence of 2,480 core genes and strain specific accessory and unique genes are given in Table 2. Strain SAMP had 130 unique genes. Pan-genome analysis revealed 89.15% core genes, 7.23% accessory and 5.34% unique genes. The numbers of shared genes were plotted as a function of number of strains (Figure 1C). The analysis of core genome led to the identification of all the COG categories and cell control, cell division and chromosome portioning also found in core genome. The COG analysis for accessory genes mostly attributed in transcription, secondary metabolite biosynthesis, transport and in catabolism category (Figure 1D). orthoMCL analysis of core genes led to the identification of 1,124 genes present in a single copy and multiple copies for other remaining genes. Functional analysis of core genes showed distribution in a varied range of KEGG categories with most of the genes associated with carbohydrate metabolism (Supplementary Figure S1). Further core genome (single gene) phylogeny was constructed to give insights into the phylogenetic relationships between the strains (Supplementary Figure S2). Functional analysis was also done for accessory and unique genes. Most of the genes in the accessory category could be assigned to the some or the other KEGG category while, 10% of them could not be assigned to any of the KEGG category and were hypothetical. The most important unique genes associated with strain DSM 17275 was cell wall binding repeat two family proteins, for strain MKU009 was DUF559 domain-containing protein and for strain SAMP was the glucose-1-phosphate thymidylyltransferase.

**Table 1:** Showing general genome features of the genome under study.

Features	*Planococcus maritimus* DSM 17275	*Planococcus maritimus* MKU009	*Planococcus maritimus* SAMP
DNA (total number of bases)	3,280,721	3,251,644	3,216,408
GC content %	47.27	47.27	47.2
Total number of genes	3,246	3,259	3,216,408
Protein coding genes	3,144	3,117	3,111
RNA genes	102	86	93
rRNA genes	27	18	26
5S rRNA	9	8	8
16S rRNA	9	6	8
23S rRNA genes	9	4	10
tRNA	71	64	63
Accession number	NZ_CP016538.2	LTZG00000000	MINM00000000


**Table 2:** Showing the number of core, accessory and unique genes within the strains.

Organism name	No. of core genes	No. of accessory genes	No. of unique genes
*Planococcus maritimus* DSM 17275	2,840	63	292
*Planococcus maritimus* MKU009	2,840	188	174
*Planococcus maritimus* SAMP	2,840	189	130


### Screening for Secondary Metabolites Genes

**FIGURE 2:**
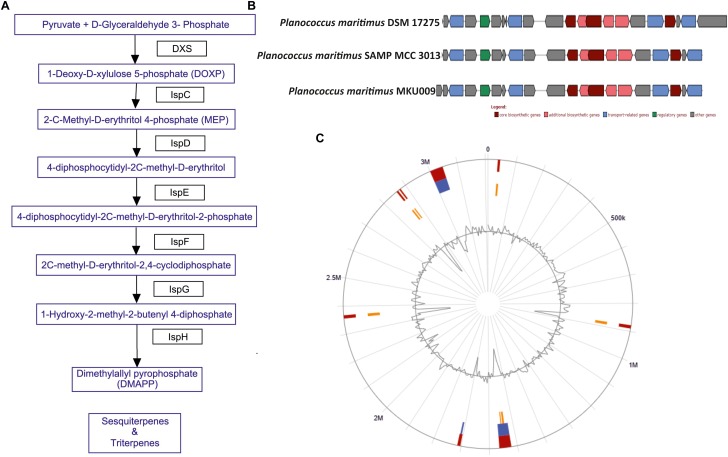
**(A)** The biosynthetic pathway found in the strain *P. maritimus* SAMP for terpenoid production. **(B)** Comparison of terpene biosynthesis gene cluster with the available genomes. **(C)** Genomic island of SAMP. Red color defines predicted genomic islands using integrated method. The blue color shows genomic islands predicted by IslandPath-DIMOB while yellow color shows genomic islands predicted by SIGI-HMM method. The broken lines represent scaffolds borders.

The screening for secondary metabolites genes were identified, a total of 33 different genes cluster were seen but only cluster 31 was the best match with highest identity of 88%. The entire cluster was further screened for the presence of secondary metabolite genes having terpene like molecule in the backbone as in lipid synthesis pathway (Supplementary Figure S3). Moreover, the entire gene sets involved in the synthesis of this terpene was identified (Figure 2A). The identified cluster was also probed in other two genomes, to know their potential for production of terpene. The gene cluster analysis for all the three strains are as shown in Figure 2B. The location of the gene cluster in all the three genomes is different, the entire gene cluster was on positive strand for two strains DSM17275 and SAMP while for strain MKU009 on the negative strand.

### Genomic Islands Analysis

Genomic islands are extensively used to compare the bacterial strains and identify vital genes in bacterial genome (Dobrindt et al., 2004; Langille et al., 2010). Mostly genomic islands associate with horizontal gene transfer (HGT) which is also known as mobile genetic elements. There are 15 genomic islands in SAMP strain that was predicted by IslandViewer 3 (Dhillon et al., 2015) and the localization of the predicted genomic islands is shown in Figure 2C. The 15 predicted genomic islands consist of 331 genes. The genes producing secondary metabolites were not found within the genomic islands.

### Determination of Surfactant Activity

**FIGURE 3:**
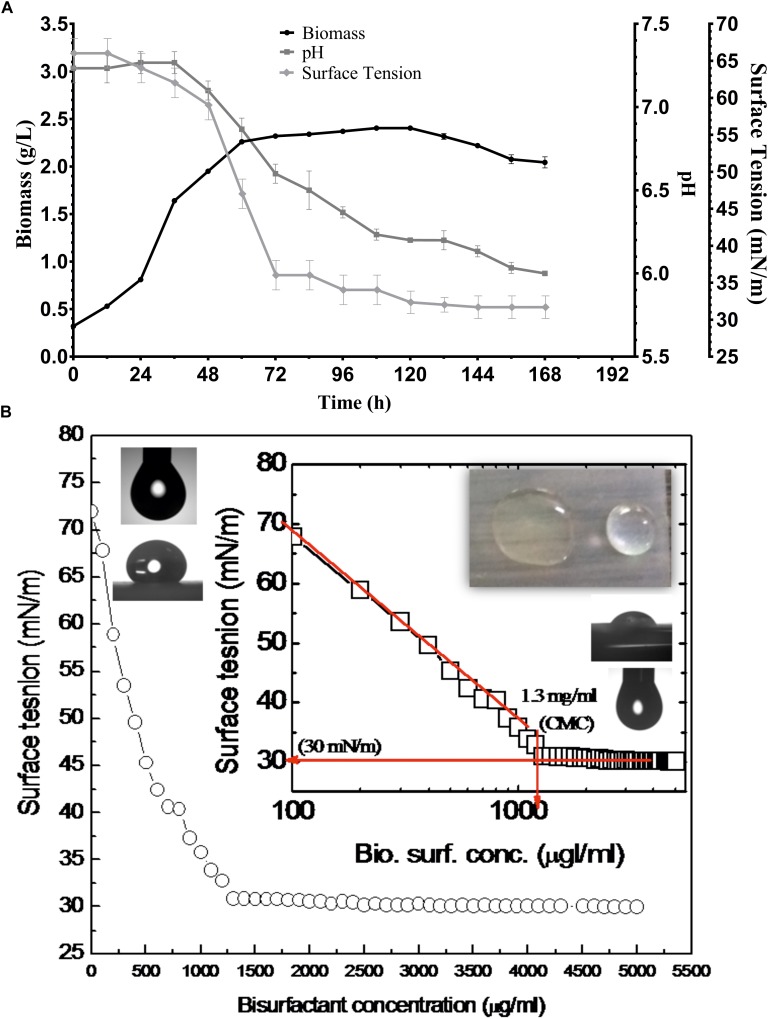
**(A)** Growth and production of biosurfactant by *P. maritimus* SAMP. **(B)** Variation in SFT inset showing semi-logarithmic reflection of CMC value of SFT (mN/m) verses concentration of biosurfactant.

Various screening procedures namely DC, OD, SFT reduction, IFT, CA and EI proved the surfactant activity of the BS obtained from *P*. *maritimus* SAMP MCC 3013. Results were comparable with positive (SDS) and negative (water) controls. The CFS and the partially purified BS had significantly displaced the oil layer and spread in the water. The production medium supplemented with 1.5% glucose was found to be suitable medium for production of BS. Figure 3A indicates the growth of *P. maritimus* SAMP was associated with the production of BS, which was assessed by observing the decrease in SFT of the medium over time. Growth of the organism was heavy during the time period of 24 to 72 h. The CFS was capable of reducing the SFT of medium from 66 to 34 mN/m. Slight increase in biomass was observed till 120 h followed by a decrease in biomass. Similarly, a slight reduction in SFT of the medium was observed till 120 h after which the SFT of the production medium remained constant (∼30 mN/m). A slight change in the pH of the media from 7.4 to 6.0 was observed during the course of the experiment (Figure 3A). The DC size of CFS was found to be 9 mm as compared with uninoculated liquid broth (5 mm).

After purification the physical properties of BS were carried out to investigate its efficacy. BS solutions effectively reduced the SFT of PBS with gradual increasing concentration of BS. The BS was capable of reducing the SFT of PBS from 70 to 30 mN/m with a CMC value of 1.3 mg/mL. The size and shape of pendant drop formed by the control (1) and BS (2) solution are shown as insets in Figure 3B. The purified BS reduced the IFT of kerosene interphase from 27 to 8 mN/m and for 2T engine oil recorded to be 22 to 7 mN/m.

### Stability Studies of *Planococcus* Derived Biosurfactant

These studies indicated the optimum stability of BS ranging between pH 6 to 8 (30 mN/m); whereas extreme acid or alkaline condition showed altered SFT values (34 mN/m) (Supplementary Table S1). Temperature treatment of above 60°C results a slight alteration in SFT values from 30 to 40 mN/m. The treatment of BS at 15% NaCl indicated optimum SFT (30 mN/m) activity. However, with increased salt concentration (>15–25%) the minor increase in SFT values were seen.

### Determination of Contact Angle (CA) and Emulsification Activity (EA)

Like SFT, IFT, a significant reduction in CA was also observed on different surfaces. The shape of the sessile drop formed by BS solution on different surfaces was compared with PBS as negative control. The maximum reduction in CA was observed on Teflon from (θ = 125.3° to θ = 103.0°, followed by Parafilm (θ = 103.9° to 91.2°) and glass (θ = 22.4°to 20.0°) surfaces. The drop formed by BS solution on Teflon surface was found to be flattened in comparison with the drop formed by control PBS. In addition to SFT, IFT, CA measurements; emulsification activities also proved its efficacy as a surfactant. BS emulsified various hydrocarbons and oils that were included in the present work. Maximum E_24_ value was observed with diesel (78%) followed by kerosene (68%), n-hexadecane (64%), toluene (56%), coconut oil (54%) and castor oil (53%). The results of all tests indicated that the purified BS had both surfactant and emulsifier properties. The synthesized BS was found to be anionic in nature; evident from the line of precipitin formed with CTAB (cationic detergent).

### Chemical Characterization

#### Thin Layer Chromatography

**FIGURE 4:**
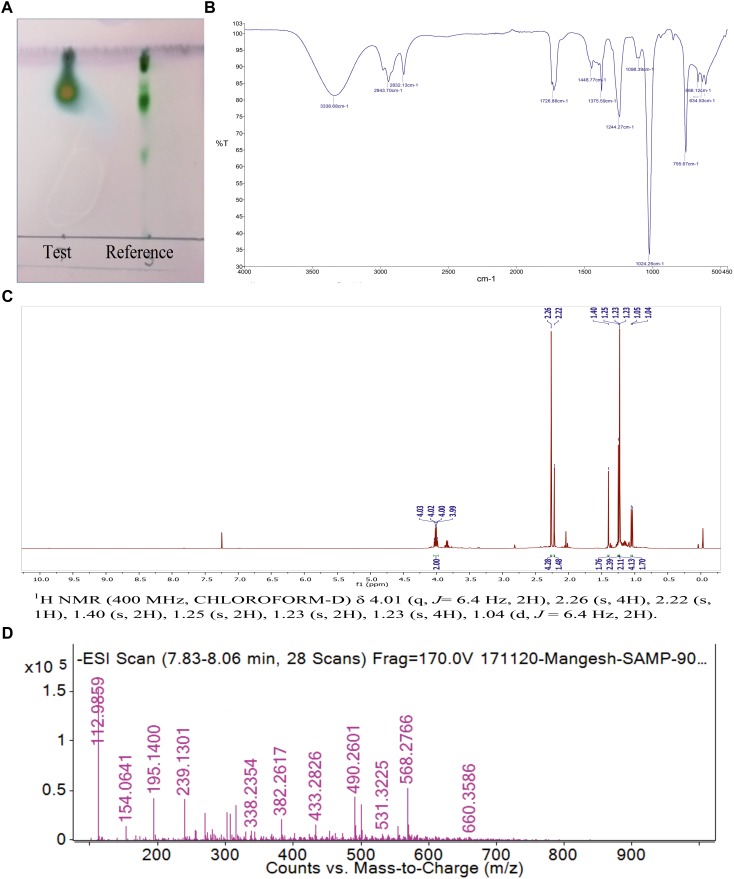
**(A)** The Thin layer chromatography (TLC) of purified biosurfactant in comparison with reference rhamnolipid. **(B)** FTIR spectrum of biosurfactant from *P. maritimus* SAMP. **(C)**^1^H NMR spectrum of biosurfactant from *P. maritimus* SAMP. **(D)** LC-MS spectrum of biosurfactant produced by *P. maritimus* SAMP.

The single yellowish green spot appeared when TLC plate was developed with anisaldehyde reagent. This confirmed the presence of glycolipid type BS with an *R*_f_ value of 0.77 (Figure 4A). The *R*_f_ values of di-rhamnolipids were noted as 0.41 (first spot) and 0.71 (second spot). The thin layer chromatograms performed to detect the presence of sugar, displayed single spot with *R*_f_ value of 0.8 which did not match with any of the reference sugars included in the experiment. The *R*_f_ value for RHL was found to be 0.7. TLC plate developed with iodine crystals showed five spots of RHL (0.6), linoleic acid (0.3), cholesterol (0.4), dipalmitin (0.6), oleic acid (0.6), and tripalmitate (0.9). The TLC plate developed with 0.2% ninhydrin reagent did not display the presence of any amino acids, confirming the sugar and lipid moieties in a BS formed by *P. maritimus* SAMP.

#### Fourier-Transform Infra-Red (FTIR)

Analysis of BS by using FTIR showed the presence of functional groups of terpenoids. The molecular composition of BS indicated the presence of most prominent absorption peaks were 3,338, 2,943, 1,726, 1,448, 1,375 and 1,024 cm^-1^. The presence of aliphatic hydrocarbon chains was seen in BS derived from *Planococcus*. Due to the presence of hydrogen bonding, a strong and broad band of the hydroxyl group (-OH); a free stretch was observed at 3,338 cm^-1^. The occurrence of C-H stretching vibrations for hydrocarbon chain of alkyl (CH_2_-CH_3_) groups was confirmed by the absorption band at 2,943 cm^-1^. Characteristic carbonyl stretching band denoted presence of ketone compounds and was found at 1,726 cm^-1^. The IR data reveals the presence of C=O, O-H and aliphatic C-H functionality in the BS produced by *Planococcus* which are major functionality of terpnoid (Figure 4B) (Yamada et al., 2015).

#### Nuclear Magnetic Resonance (NMR)

The ^1^H and ^13^C NMR spectra of *Planococcus* BS showed the presence of terpenoids. The presence of a terpene described by the peak detected at δ 1.04 and δ 1.40 ppm for terminal methyl group and methylene group, respectively. Additional peaks at δ 1.23–1.4 indicated the methyl (–CH_3_) group. The peaks found at δ 4.03–3.82 for two protons displayed the presence of two –CH_2_– groups (Figure 4C). The ^13^C NMR analysis demonstrated a peak of carbonyl carbon at δ 211.67 ppm and the peak at δ 80 ppm indicated the presence of carbon with oxygen bond. The peak observed at δ 15–20 ppm shows methyl carbon peak indicating the presence of –CH_3_ group (Supplementary Figure S4). The NMR data is in good agreement with IR data that supports the presence of terpenoid like molecules. The spectral characteristics achieved for BS (other than *Planococcus*) were in good accordance with the data published in the previous literature (Kügler et al., 2015; Aleksic et al., 2017).

#### Liquid Chromatography-Mass Spectrometry (LC-MS/MS)

Thin layer chromatography was further deciphered by mass spectrometry. LC-MS analysis of the BS was performed in negative ion mode. LC-MS data described the major ion peak of molecular mass m/z 195.14 (Figure 4D) were observed predominantly as the terpenes produced by marine bacteria (4R,5Z)-dodec-5-en-4-olide (De Carvalho and Fernandes, 2010).

Chemical characterization using TLC, FT-IR, NMR and LC-MS found that the indigenous strain *P. maritimus* SAMP produces BS having sugar and lipid moieties. Therefore, the term ‘terpene containing BS’ is denoted to the BS produced by *Planococcus* sp. Therefore, we speculate that terpene BS produced by *Planococcus* have significant contribution in the field of BS produced by diverse marine microbes.

## Discussion

Halophiles, an interesting class of ‘extremophilic organisms’ have been acknowledged for production of diverse types of stable, unique bio-molecules like BS (DasSarma and Arora, 2002) having great stability at high temperature, pH and different salt concentrations. The interesting functional properties of BS viz. emulsification, wetting, foaming, cleansing, surface activity and reduction in viscosity of crude oil makes them suitable competitors for industrial applications (De et al., 2015). Therefore, search for BS producing; indigenous, potent microorganism is an imperative area of research. A variety of marine environments (solar saltern, deep sea, sea floor, salt marshes, estuaries, intertidal zones, mangroves, lagoons, coral reefs) are dominated with *Planococcus* sp. (Engelhardt et al., 2001). This aerobic and heterotrophic bacterium can degrade various hydrocarbons and therefore can contribute significantly to reduce contamination of hydrocarbon in marine environments (Engelhardt et al., 2001; Kumar et al., 2007). The foremost statement documented by Kumar et al. (2007) reported *Planococcus* sp. derived EPS having high emulsifying and tensiometric properties making them useful product for bioremediation and microbial enhanced oil recovery (MEOR). Work contributed by Ebrahimipour et al. (2014) documented the production of glycolipid type BS from *Planococcus* sp. Thus from the literature *Planococcus* strains appears to be as one of the promising microbial system which can be exploited for production of surface active agents.

The marine bacterium isolated (from sea shore of Ratnagiri, Maharashtra, India) by us was identified as *P. maritimus* through genome sequencing. This analysis of *P. maritimus* SAMP revealed complete genes in the pathway for the synthesis of terpenoid, which could be the backbone molecule for production of BS (Figure 2A). The genes in the pathway were not located in the genomic islands thus suggesting the inherent potential of *Planococcus* sp. to synthesize the terpenoid molecule. We would like to suggest that the later stages of terpene biosynthesis might be diverted to BS synthesis. Detailed outlook on synthesis of BS warrants further investigation. The comparative genome analysis has helped us to identify the genomics potential of all the three strains and confirmed 2,840 core genes. The presence of terpene synthesis cluster was observed in all three genomes, but insertion sequence elements (IS) were associated with DSM 17275 before the start of the cluster. Further studies are mandatory to verify the production capacity of BS from other two strains. Moreover, these genes were present in the core genome, suggesting its inherent potential for terpene biosynthesis.

The morphological feature including the short cocci of Gram-positive bacterium was described successfully for SAMP with the help of SEM. The phylogenic analysis of ribosomal RNA gene sequence indicated the member belonging to the genus *Planococcus* (99%). Confirmation of signature nucleotides of *Planococcus* was well supported by the 16S rRNA gene sequencing (See-Too et al., 2017). Our investigation on genomic insight of BS biosynthetic pathway suggests the involvement of terpenoid type products. Terpenoids are popularly known as antimicrobial, anti-inflammatory and chemotherapeutic agents having pharmaceutical significance (Thoppil and Bishayee, 2011). The mevalonate pathway and the non-mevalonate (non-MVA) pathways are involved in the production of terpenoid precursors (Hunter, 2007). Based on our observation we propose that the genus *Planococcus* produces terpene containing BS through non-mevalonate pathway. It has been suggested that the terpene synthases are mainly responsible in contributing the diversity as well as complexity of the metabolites produced by the microbial system (Abdallah and Quax, 2017).

We used screening method to detect BS production by the strain MCC 3013 and culture was found to be positive in DC, OD assay and in EI. From the literature, it appears that all three techniques have been preferred usually to identify BS producers. Our results were supportive to our previous published literature (Satpute et al., 2008). Media composition plays a vital role in determining the nature and chemical composition of the BS produced by microorganism. It is evident from the literature that production medium supplemented with different carbon sources promotes the production of BS from *Planococcus* sp. (Engelhardt et al., 2001; Kumar et al., 2007; Ebrahimipour et al., 2014). We have successfully demonstrated the BS production from *P. maritimus* SAMP in production medium supplemented with glucose (1.5%). Extraction of the BS product was carried out after 1 week of incubation. Being a marine bacterium, the maintenance of optimal salt concentration is necessary for the growth and production of BS. One of the possible reasons for this could be the switching off some important gene/s involved in the metabolic pathway of BS production. It has been reported that the carbon: nitrogen ratio is a rate- determining factor in BS production (Santos et al., 2016). Surface area is the critical parameter which needs to be taken care of, during the BS fermentation processes (Satpute et al., 2016). The production of BS by SAMP strain showed direct relationship with cell growth, i.e., BS accumulated in the production medium (supplemented with 1.5% glucose) as the cells entered into the exponential phase of their growth. The SFT of fermentation medium was reduced significantly from 66 to 32 mN/m within 168 h of incubation indicated the production of BS maximum by the end of growth phase (Figure 3A). The decrease in SFT of the medium was drastic during the same time period, representing the association of BS production along with growth of the organism. The spreading of the drop on the parafilm coated surface supported the previous observation. A parallel relationship between the utilization of medium components, growth and BS production is usually growth-associated. We agree with this opinion previously placed by Ebrahimipour et al. (2014) for *Planococcus* sp. and for other microorganisms (Chen et al., 2006; Ebrahimipour et al., 2014).

The demand of microbial originated surfactant is steadily increasing and therefore there is need to improve the detection methods, production and purification protocols (Satpute et al., 2010b; Banat et al., 2014). Work through such diversified techniques authenticates the presence of commercial important products. The effectiveness of purified BS is determined by its low CMC value and the ability to reduce SFT and IFT. A good surfactant can reduce the SFT of water from 72 to the lower ranges. A well-known chemical surfactant, i.e., SDS reduces SFT of water significantly (Stark et al., 2014). Kumar et al. (2007) have reported that the *Planococcus* sp. can reduce the SFT of culture broth from 72 to 46.07 mN/m. However, in our study with *P. maritimus* SAMP, the purified BS effectively reduced the SFT of fermentation broth from 65 to 35 mN/M and for PBS from 70 to 30 mN/m with a CMC of 1.3 mg/mL. Nevertheless, this is the first report providing detailed investigation on physical properties of *Planococcus* derived BS. The terpene containing BS is also effective at oil water interfaces (evident from the IFT measurements). The stability studies at different pH, temperature and salt concentration resulted appreciable properties of terpene containing BS and therefore proved the properties of ideal surfactant. One of the significant finding of this study is thermo-stability of the BS produced by *P. maritimus* SAMP. The BS produced by *Planococcus* was stable even after autoclaving. Such extreme temperature stability was reported for BS isolated from *Pseudomonas aeruginosa* strain (Abdel-Mawgoud et al., 2008). The thermal stability of the BS increased the scope of its application in MEOR where high temperature is prevailed. Due to these extraordinary properties of BS; their commercial demand is enormously increasing (Patowary et al., 2017). To meet this demand, our search toward potential microbial product becomes imperative.

Several researchers have used different combinations of solvent systems to extract BS from microorganisms. We found chloroform: methanol (2:1, v/v) as suitable solvent system for extraction of BS. We state that column chromatography is an essential methods to obtain the microbial products in purified form. This is in an agreement with our recent published work (Hamza et al., 2017). Characterization through analytical techniques like, TLC, FTIR, NMR and LC-MS made it feasible to disclose the chemical nature of BS. Complete authentication of functional groups with lipid, carbohydrate moieties and absence of protein fractions is in agreement with Ebrahimipour et al. (2014). Previous literature on RHL illustrates that the presence of fatty acid chains and carbohydrate moieties in BS (Abdel-Mawgoud et al., 2009; Abbasi et al., 2012; Aleksic et al., 2017). Our NMR studies also proved the presence of terpene moieties in a BS which is in agreement with Kügler et al. (2015). In addition to NMR analysis, the structural composition was identified through LC-MS to illustrate the presence of peaks of molecular masses corresponding to the members of the lipid and polysaccharide. Thus analytical characterization of BS through above mentioned techniques described occurrence of sugar and lipid moieties. Similar kind of interpretation has been proposed for BS (other than *Planococcus* sp.) by Wang et al. (2014) and Patowary et al. (2017). The term glyco-carotenoids/-terpenoids has been used to indicate BS derived from *P. maritimus* (Abdel-Mawgoud and Stephanopoulos, 2017). Basically terpenes are hydrocarbons containing molecules. Our observations are in agreement with the recently published literature by Abdel-Mawgoud and Stephanopoulos (2017). Based on above findings, we propose that the indigenous marine bacterium *Planococcus* is a powerful source for the production of terpene containing BS which could have noteworthy applications.

## Conclusion

Our combined attempts on purposeful genomics offer a partial chemical and a genetic leads to illustrate the biochemical depiction of a bioactive compound (BS) produced by *P. maritimus*. In this report, *P. maritimus* was confirmed as BS producing strain in a fermentation medium supplemented with glucose as carbon source. Based on the physical properties like SFT, IFT, CMC, CA and EI, we suggest that SAMP as one of the ingenious BS producers. From genome analysis, we traced out the biosynthetic pathway for terpene. However, the later stages of terpene biosynthesis might be diverted toward BS secretion; detailed investigation on this aspect would be helpful to prove this possibility. Terpene containing BS significantly reduced the SFT of PBS from 72 to 30 mN/m with a CMC value of 1.3 mg/mL. Systematic investigation on *Planococcus* BS through TLC, FT-IR, NMR and LC-MS disclosed the presence of sugar and lipid moieties. Perhaps this is the first report documenting the detailed investigation on physico-chemical properties on BS of *Planococcus* origin. Based on genomic and functional analysis, the term terpene containing BS is denoted for the surfactant produced by *P. maritimus*. We believe that the production of BS from *Planococcus* would be recognized in the literature in the parallel line of previous popular BS (Surfactin-*Bacillus* sp.; Surlactin-*Lactobacillus* sp.; Rhamnolipid-*Pseudomonas* sp.). The foremost intention on evaluation of functional properties would definitely enrich the literature on BS producing *Planococcus* sp.

## Author Contributions

SW, MS, and SS conceived and designed the experiments. SW, SK, PD, AB, and SS performed the experiments. All authors analyzed the data and wrote the manuscript.

## Conflict of Interest Statement

The authors declare that the research was conducted in the absence of any commercial or financial relationships that could be construed as a potential conflict of interest.

## Supplementary Material

Figure S1 KEGG distribution in core, accessory and unique genomes.

Figure S2 Core genome based phylogeny of *P. maritimus* strains.

Figure S3 Glycolipid biosynthesis pathway driven from KEGG annotations to *P. maritimus* SAMP genome.

Figure S4 ^13^C NMR spectrum of biosurfactant from *P. maritimus* SAMP.

Table S1 Stability studies of *Planococcus* derived biosurfactant with different pH, temperature and salt concentration.
