# Supplementary figures and images for: Genomic Insights of Halophilic Planococcus maritimus SAMP MCC 3013 and Detail Investigation of Its Biosurfactant Production
Source: Front Microbiol. 2019 Feb 26;10:235. doi: 10.3389/fmicb.2019.00235 (PMC6399143; doi:10.3389/fmicb.2019.00235)

# KEGG Distribution

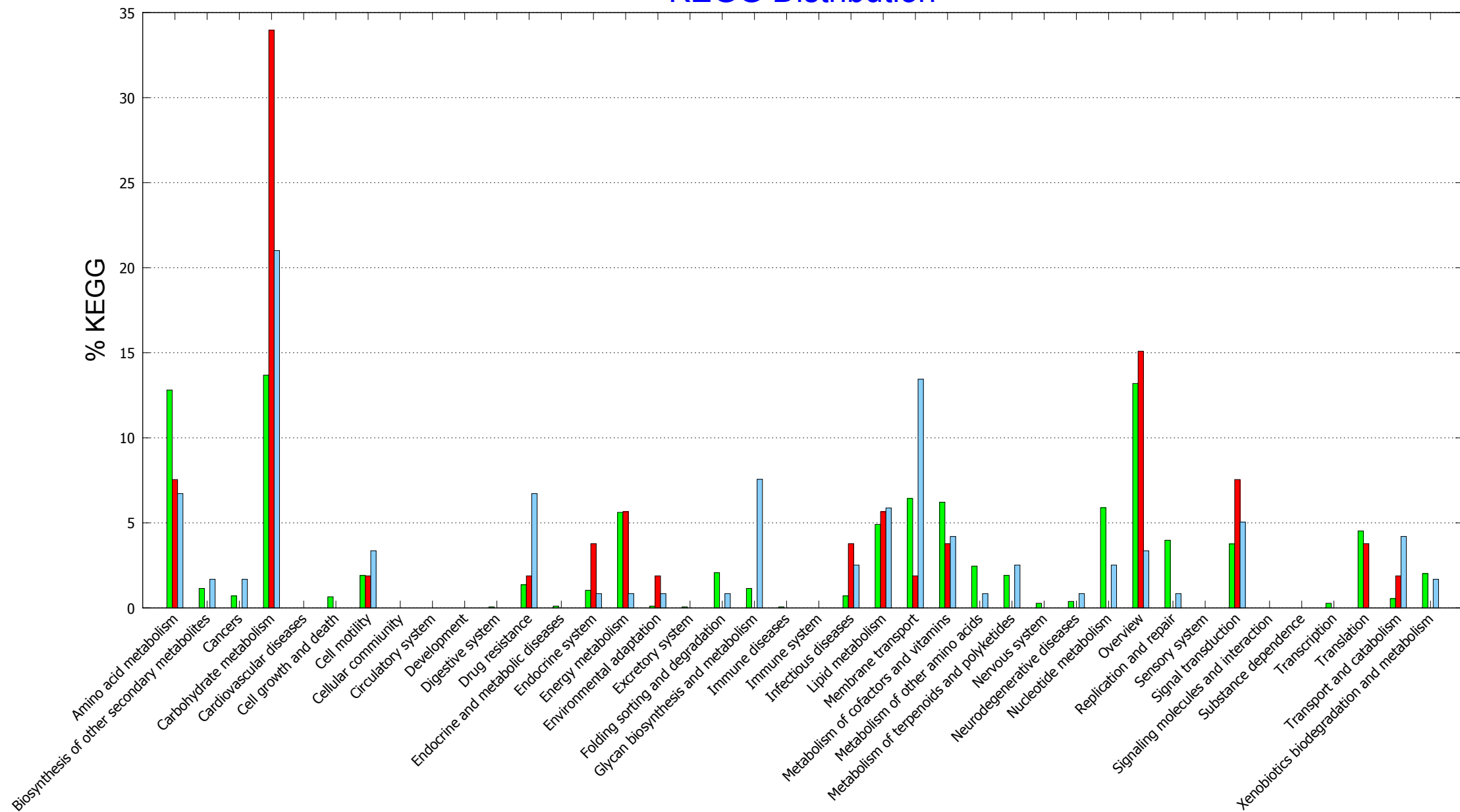

CORE  
ACCESSORY  
UNIQUE

Supplement: Figure S1 — KEGG distribution in core, accessory and unique genomes. [file Image_1.pdf]

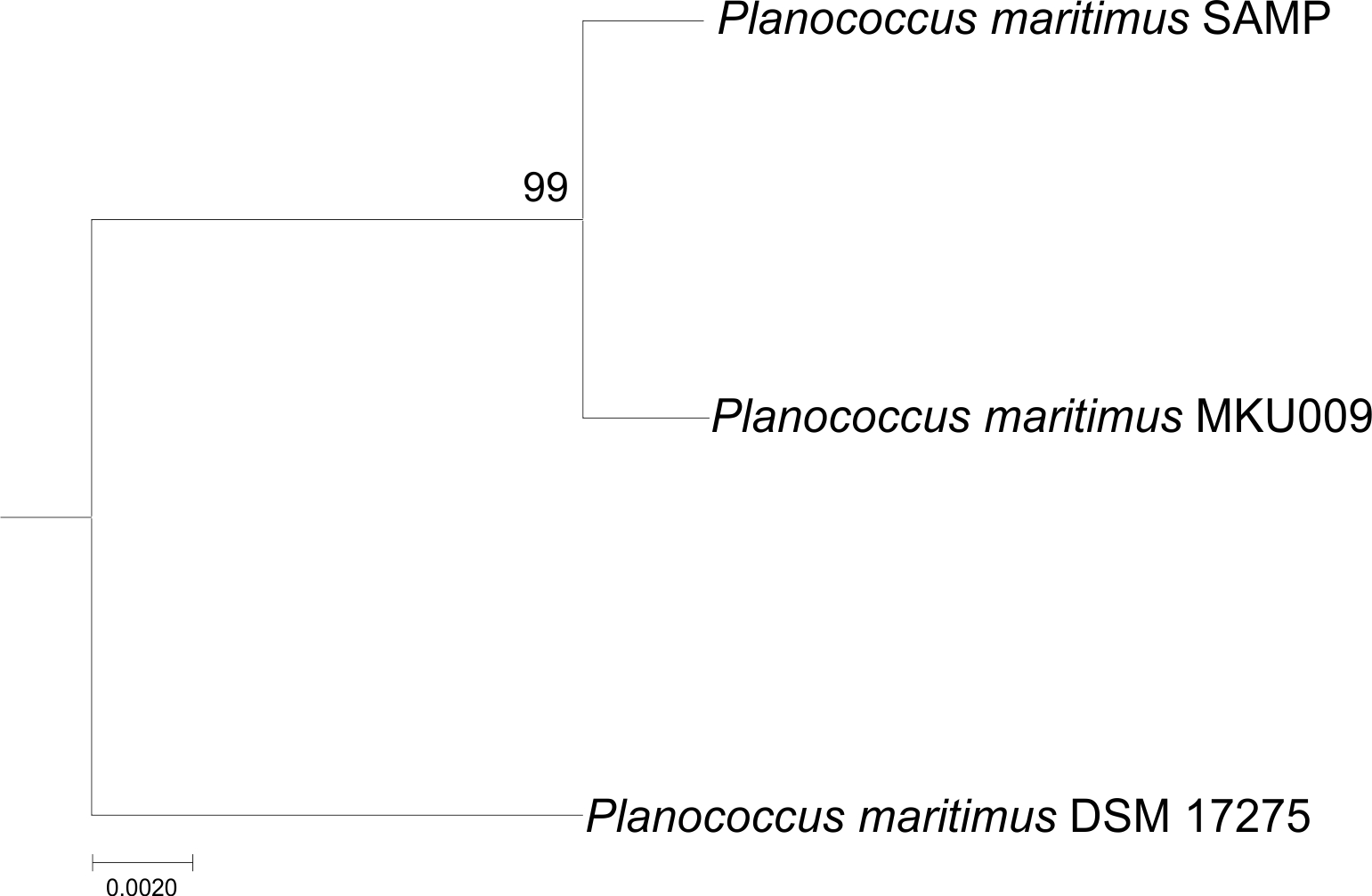

Supplement: Figure S2 — Core genome based phylogeny of P. maritimus strains. [file Image_2.jpeg]

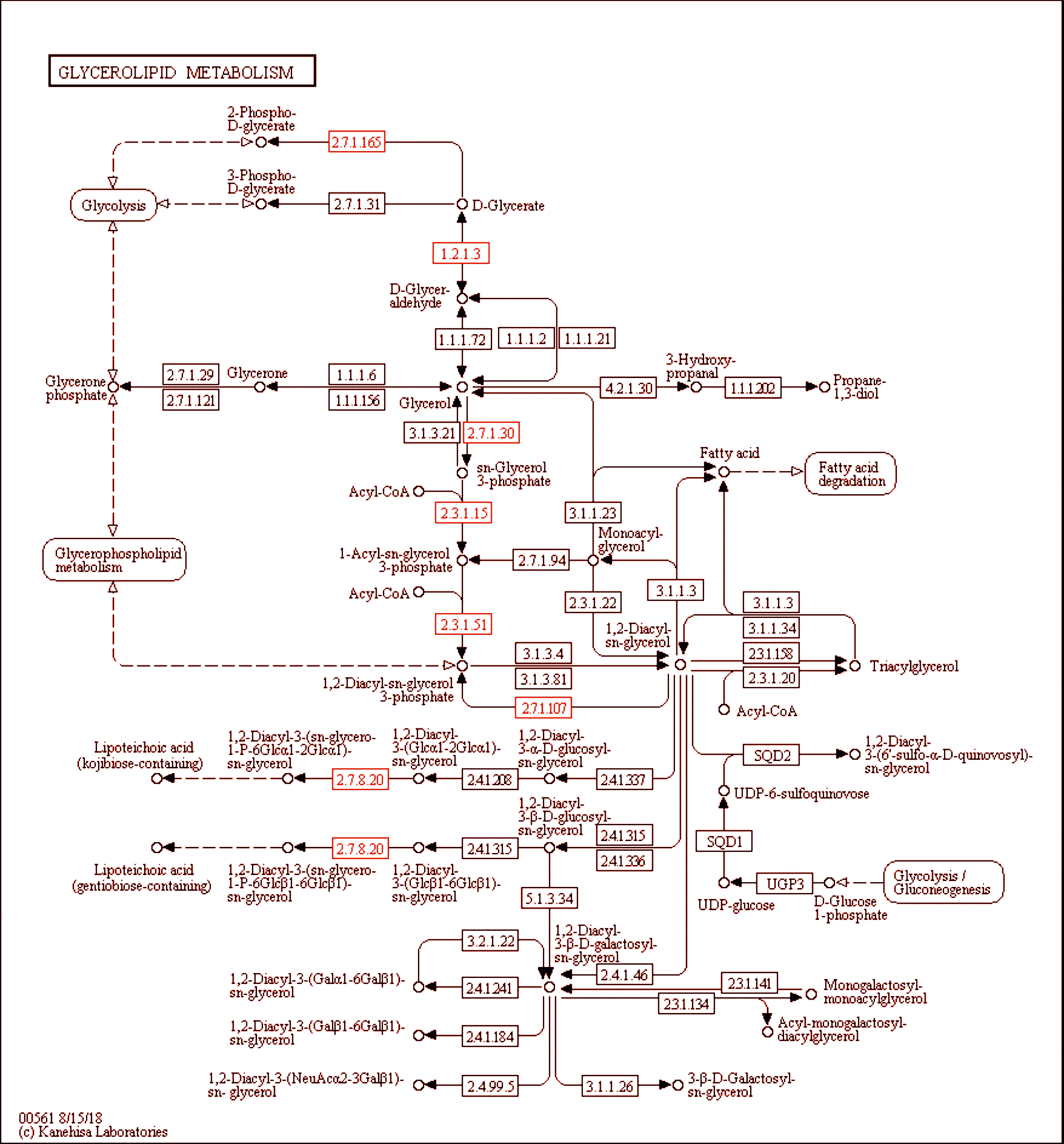

Supplement: Figure S3 — Glycolipid biosynthesis pathway driven from KEGG annotations to P. maritimus SAMP genome. [file Image_3.jpeg]

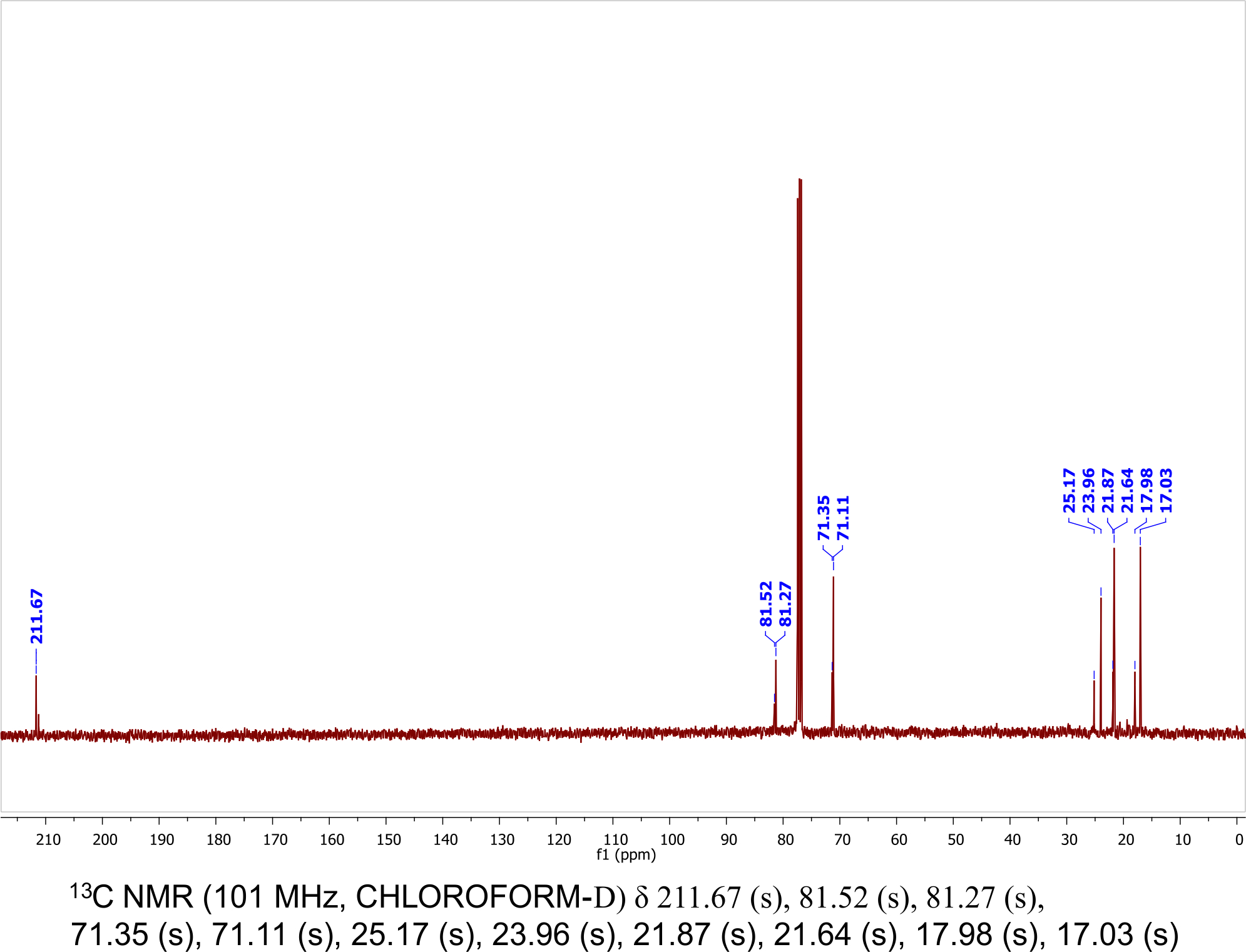

Supplement: Figure S4 — 13C NMR spectrum of biosurfactant from P. maritimus SAMP. [file Image_4.jpeg]
