# Supplementary material for: Genomic Insights of Halophilic Planococcus maritimus SAMP MCC 3013 and Detail Investigation of Its Biosurfactant Production
Source: Front Microbiol. 2019 Feb 26;10:235. doi: 10.3389/fmicb.2019.00235 (PMC6399143; doi:10.3389/fmicb.2019.00235)
Supplement: Table S1 — Stability studies of Planococcus derived biosurfactant with different pH, temperature and salt concentration. [file Table_1.DOCX]

| **Parameters** | **Surface Tension*** **mN/m** |
| --- | --- |
| **pH** | |
| 2 | 34 |
| 4 | 32 |
| 6 | 30 |
| 8 | 30 |
| 10 | 32 |
| 12 | 34 |
|  | |
| **Temperature °C** | |
| 20 | 30 |
| 40 | 30 |
| 60 | 30 |
| 80 | 34 |
| 100 | 38 |
| 120 | 40 |
|  | |
| **NaCl (%)** | |
| 0 | 30 |
| 5 | 30 |
| 10 | 30 |
| 15 | 30 |
| 20 | 32 |
| 25 | 34 |

*Mean values
